# Aminoglycoside Antibiotics Inhibit Mycobacteriophage Infection

**DOI:** 10.3390/antibiotics9100714

**Published:** 2020-10-19

**Authors:** Zheng Jiang, Junwei Wei, Yunxiang Liang, Nan Peng, Yingjun Li

**Affiliations:** State Key Laboratory of Agricultural Microbiology, College of Life Science and Technology, Huazhong Agricultural University, Wuhan 430070, China; 1021644470@webmail.hzau.edu.cn (Z.J.); weijw@webmail.hzau.edu.cn (J.W.); liangyunxiang@mail.hzau.edu.cn (Y.L.)

**Keywords:** antibiotic resistance, tuberculosis, phage therapy, aminoglycosides, mycobacteriophage

## Abstract

Antibiotic resistance is becoming the biggest threat to global health. At the same time, phage therapy is witnessing a return of interest. The therapeutic use of bacteriophages that infect and kill bacteria is a suitable strategy to combat antibiotic resistance. Furthermore, bacteriophages are increasingly used in combination with standard antibiotics against drug-resistant pathogens. Interestingly, we found that the engineered mycobacteriophage phAE159 and natural phage D29 cannot infect the *Mycobacterium tuberculosis* in the presence of kanamycin, hygromycin or streptomycin, but the phage infection was not affected in the presence of spectinomycin. Based on a series of studies and structural analysis of the above four aminoglycoside antibiotics, it could be speculated that the amino sugar group of aminoglycoside might selectively inhibit mycobacteriophage DNA replication. Our discovery that broad-spectrum antibiotics inhibit phage infection is of great value. This study will provide guidance for people to combine phage and antibiotics to treat *M. tuberculosis*.

Bacterial infection refers to the invasion of a host’s tissue by pathogenic bacteria. Generally, antibiotics are the preferred antibacterial agents [1]. However, bacteria can evolve resistance to antibiotics resulting from antibiotics abuse and natural evolution. More seriously, bacteria are developing resistance to antibiotics in an increasingly fast manner compared with the slow development of antibiotics over the past few decades [2]. Some pathogens have even become superbugs, and there are no effective antibiotics to fight against them [3]. Therefore, there is an urgent need to develop alternative therapies against bacterial pathogens. Among the many emerging antibacterial strategies, phage therapy is considered a very promising solution. Bacteriophages (phages) are the viruses of bacteria, which were discovered over 100 years ago [4]. They are extremely widespread in nature, with an estimated number of approximate 10^31^, far more than other microorganisms [5,6]. Since then, phages have been employed clinically to treat bacterial infections as a natural antimicrobial [7,8]. Phage therapy has been proven to be efficacious in at least one modern efficacy (phase I/II) clinical trial [9], and there are some successful cases of applying phages to the treatment of human drug-resistant bacterial infections [10,11,12,13,14]. Bacteriophages provide an alternative useful antibacterial approach and have gradually been used in combination with standard antibiotics against the drug resistance of pathogenic bacteria, which is mainly based on the phage–antibiotic synergy (PAS) effect [15]. The phage–antibiotic synergy effect refers to the phenomenon that the sub-lethal concentrations of certain antibiotics can significantly stimulate the propagation of lytic bacteriophages in the host bacteria, leading to accelerated cleavage of host cells and rapid diffusion of progeny phages [15], and the co-treatments of antibiotics and phages can achieve better therapeutic effects compared with single treatments. The problem to be solved in particular is whether antibiotic treatment has an antagonistic effect on the activity of phage infection, especially in the treatment of antibiotic concentrations greater than or equal to MIC (Minimal Inhibitory Concentration) for targeting bacteria that are clinically sensitive to antibiotics [16]. An important question arises: Do antibiotics impact the pharmacodynamics of phage therapy?

Tuberculosis (TB) is a chronic infectious disease caused by *Mycobacterium tuberculosis*, with around 10 million people falling ill with TB each year. Tuberculosis accounts for the highest mortality from any infectious diseases worldwide, even surpassing HIV/AIDs, causing 1.5 million deaths in 2018 [17]. Drug-resistant bacteria occur frequently due to the abuse of antibiotics, which seriously threaten public health and social safety. Tuberculosis is the world’s leading cause of death from infectious diseases, and the drug-resistant form of the disease is a major risk to global health security. In 2016, there were an estimated 10.4 million new tuberculosis cases globally, and 600,000 new cases of resistance to rifampicin, the most powerful first-line drug [18]. New treatment methods are urgently needed because existing anti-tuberculosis drugs can no longer meet the need for the treatment of drug-resistant TB [19]. Our primary goal is to use the endogenous CRISPR-Cas system to eliminate *Mycobacterium tuberculosis* by engineering mycobacteriophage. In order to avoid the contamination of microbes in an infection test, we used a *Mycobacterium tuberculosis* strain that carries a plasmid with a kanamycin-resistance gene and added kanamycin to the culture. Interestingly, we found that the addition of kanamycin in the culture greatly reduced the ability of the engineered mycobacteriophage to infect the host strain. Then, we examined the ability of kanamycin to inhibit the infection of other mycobacteriophages, including the TM4-derived phasmid phAE159 [20] and phage D29. Similar to previous results, the two phages were able to form plaques on the plates of *M. smegmatis* mc^2^155 in the absence of kanamycin. In the presence of the above antibiotics, the replication of the two mycobacteriophages was inhibited by 10^3^ folds or more (Figure 1a). We determined the inhibitory concentration of kanamycin to phage D29 to be 50 μg/mL, and this concentration did not affect the growth of *M. smegmatis* mc^2^155 (Figure S1). Furthermore, we verified the inhibitory effect of kanamycin on the infection test of *M. tuberculosis* H37Ra and *M. bovis* BCG by phage D29 (Figure S2). This demonstrates that kanamycin can suppress the infection ability of the mycobacteriophage phAE159 and D29.

To determine whether other bacteriophages can also be suppressed by kanamycin, we examined the ability of kanamycin to protect *Escherichia coli* from lysis by the well-characterized dsDNA phages T7 and λ. The plaque and growth curve assays indicated that kanamycin did not affect the *E. coli* phage infection (Figure 1b), suggesting that this inhibitory effect might be specific to mycobacteriophages. In addition, we investigated the effect of another antibiotic hygromycin commonly used in the study of *Mycobacterium*, which is also an aminoglycoside antibiotic. The results indicated that the hygromycin was able to effectively inhibit mycobacteriophages phAE159 and D29 (Figure 1c). Subsequently, we examined the ability of kanamycin to suppress *M. smegmatis* lysis by the mycobacteriophage vector phAE159 through electroporation. The plaques were observed in the absence but not in the presence of kanamycin (Figure 1d). 

In order to determine whether the antibiotic directly acts on the phages, such as by destroying the phages, *M. smegmatis* mc^2^155 was pre-incubated with mycobacteriophage phAE159 with or without kanamycin and then plated on 7H10 with or without kanamycin. For each treatment, the plate was observed to be full of *M. smegmatis* after 2-day growth regardless of the addition of kanamycin during pre-incubation, and as long as the kanamycin was present in the solid medium in plates, the bacteria were not lysed by the phage and grew well (Figure 1e). This means that kanamycin may not be able to directly act on mycobacterial phage, but it can exert its inhibitory effect after the phage enters the host.

The two antibiotics we used earlier were aminoglycosides. Therefore, we further examined whether this inhibitory effect was applicable to other aminoglycoside antibiotics. First, we constructed a plasmid pSTR1 carrying *aadA* (encoding aminoglycoside adenylyltransferase) gene to confer *M. smegmatis* mc^2^155 resistance to streptomycin and spectinomycin. The antibiotic sensitivity test showed that *M. smegmatis* mc^2^155 harboring the recombinant plasmid tolerated streptomycin and spectinomycin well, while the WT did not grow in the presence of either of these two antibiotics (Figure 1f). Next, propagation assays were performed to explore the effect of these two antibiotics on the mycobacteriophage infection. The results indicated that the propagation of phage on the plate was significantly inhibited in the presence of streptomycin, but it was not inhibited in the presence of spectinomycin (Figure 1g).

In order to explore the mechanism more intuitively, transmission electron microscope (TEM) analysis was performed to determine whether the adsorption capacity of mycobacteriophage was normal in the presence of antibiotics. Before microscopic observation, we mixed phage D29 and *M. smegmatis* mc^2^155 for 15 min with or without streptomycin. The addition of streptomycin was found to have no effect on the phage morphology, indicating that streptomycin could not directly act on the phage. In addition, streptomycin did not inhibit the adsorption of mycobacteriophage to the cell surface of *Mycobacterium,* which was consistent with the control group (Figure 1h). After the bacteriophage was adsorbed to the surface of the bacteria, the injection and cyclization of its genome DNA were a very rapid process. Based on these, we speculated that the inhibitory effect of antibiotics on phage infection might occur after viral genome injection. Afterward, we used absolute quantification PCR to detect the content of phage DNA during the infection process. Bacteria were cultured in the presence of streptomycin, with mycobacteriophages added at a high multiplicity of infection (MOI), and the contents of phage DNA in culture and supernatant were detected every hour. In the absence of streptomycin, phage DNA was increased exponentially after one-hour co-culture, whereas the phage DNA was not increased in the presence of streptomycin. The same change trend in the content of phage DNA was also observed in the host cells, indicating that streptomycin inhibited the infection ability of mycobacteriophage by blocking phage DNA replication (Figure 1i). 

The life cycle of a phage starts with the adsorption and injection of the genome onto/into the host cell. After DNA injection, the phage genome is circularized, and then the DNA is replicated and assembled; finally, the host cell is lysed, and the progeny phages are released. In summary, in this study, morphology observation through TEM indicated that the aminoglycoside antibiotics did not prevent the phage from adsorbing onto the host cell. The experiment results of electroporation and pre-incubation of mycobacteriophage phAE159 and *M. smegmatis* mc^2^155 with or without kanamycin showed that the inhibition of kanamycin on phage infection occurred after the phage injected DNA into the host cell. Moreover, we observed that the mycobacteriophage DNA did not proliferate in host cells in the presence of streptomycin. Taken together, our experiment results indicated that aminoglycosides were able to block phage DNA replication and allowed the bacteria to survive (Figure 1j).

Although the antibiotics used in our study belong to aminoglycosides because they all contain amincyclic alcohols, spectinomycin differs from the three other antibiotics in that it does not contain an amino sugar group (Figure S3). Based on the above observations and results, we speculate that the possible mechanism might lie in that amino sugar group of aminoglycoside which might selectively inhibit mycobacteriophage DNA replication. Therefore, further exploration of their action mechanisms will expand our knowledge of bacterial anti-phage defense systems [21] and the battle between bacteria and phage foes [22]. For the treatment of resistant bacteria infection, phage therapy has become a backup option. Among them, phage combined with antibiotics is a commonly used strategy. Whether some antibiotics have an antagonistic effect on phages is worth investigating. In addition, it should be particularly noted that streptomycin is a first-line drug for tuberculosis, and our discovery might shake the previous understanding of the synergy between phage therapy and antibiotic therapy [16]. This study will provide guidance for people to combine phage and antibiotics to treat *M. tuberculosis*.

## Figures and Tables

**Figure 1:**
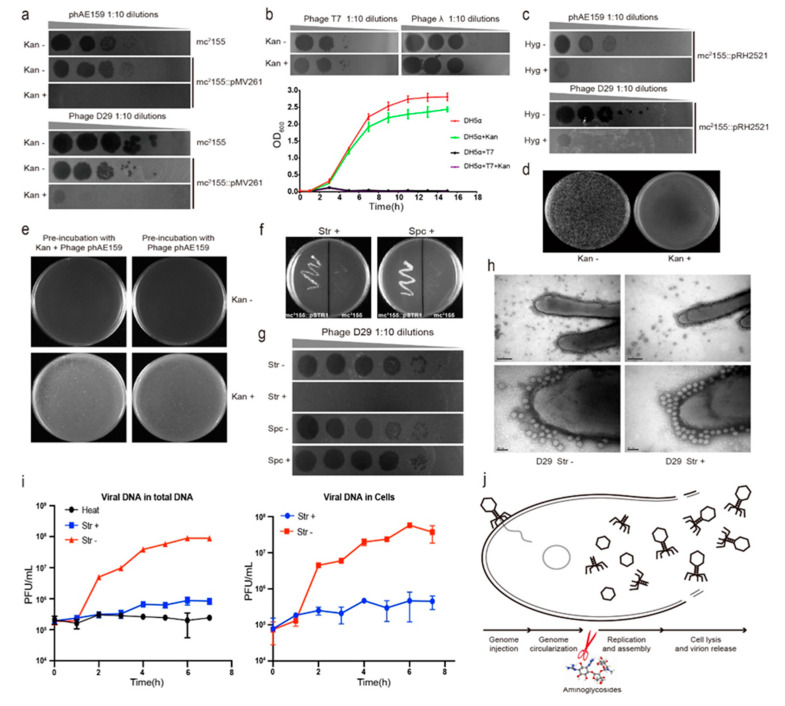
Aminoglycoside antibiotics inhibit the DNA replication of mycobacteriophages. (**a**) The replication of the two mycobacteriophages phAE159 and D29 were inhibited by kanamycin on the lawns of *M. smegmatis* mc^2^155. (**b**) Kanamycin did not affect the infection of *Escherichia coli* phages T7 and λ. (**c**) Hygromycin was able to inhibit the infection of mycobacteriophages phAE159 and D29. (**d**) Kanamycin inhibited the formation of plaques by electrotransformation of phAE159 vector. (**e**) Pre-incubation with kanamycin did not affect the infection of mycobacteriophage phAE159. (**f**) *M. smegmatis* mc^2^155 harboring the recombinant plasmid tolerated streptomycin and spectinomycin well. (**g**) The propagation of mycobacteriophage D29 was significantly inhibited in the presence of streptomycin but had no effect in the presence of spectinomycin. (**h**) TEM analysis of the adsorption capacity of mycobacteriophage in the presence of streptomycin. (**i**) Quantitative PCR analysis of phage DNA proliferation during the infection process. (**j**) Aminoglycosides were speculated to be able to block the DNA replication during the life cycle of phage (adapted from Figure 3a in [21]).

## Supplementary Materials

The following are available online at https://www.mdpi.com/2079-6382/9/10/714/s1: Figure S1: Effects of different concentrations of kanamycin on the infection of *M. smegmatis* mc^2^155 by phage D29; Figure S2: Kanamycin inhibits the infection of *M. tuberculosis* H37Ra and *M. bovis* BCG by phage D29; Figure S3: The structures of the four aminoglycoside antibiotics used in this study.

## References

[1] Kohanski M.A., Dwyer D.J., Hayete B., Lawrence C.A., Collins J.J. (2007). A common mechanism of cellular death induced by bactericidal antibiotics. Cell.

[2] Lewis K. (2020). The Science of Antibiotic Discovery. Cell.

[3] Dodds D.R. (2017). Antibiotic resistance: A current epilogue. Biochem. Pharmacol..

[4] Dublanchet A., Fruciano E. (2008). A short history of phage therapy. Med. Mal. Infect..

[5] Clokie M.R., Millard A.D., Letarov A.V., Heaphy S. (2011). Phages in nature. Bacteriophage.

[6] Mushegian A.R. (2020). Are There 10^31^ Virus Particles on Earth, or More, or Fewer?. J. Bacteriol..

[7] Abedon S.T. (2017). Bacteriophage Clinical Use as Antibacterial “Drugs”: Utility and Precedent. Microbiol Spectr.

[8] Abedon S.T. (2019). Use of phage therapy to treat long-standing, persistent, or chronic bacterial infections. Adv. Drug Deliv Rev..

[9] Wright A., Hawkins C.H., Änggård E.E., Harper D.R. (2009). A controlled clinical trial of a therapeutic bacteriophage preparation in chronic otitis due to antibiotic-resistant *Pseudomonas aeruginosa*; a preliminary report of efficacy. Clin. Otolaryngol.

[10] Bao J., Wu N., Zeng Y., Chen L., Li L., Yang L., Zhang Y., Guo M., Li L., Li J. (2020). Non-active antibiotic and bacteriophage synergism to successfully treat recurrent urinary tract infection caused by extensively drug-resistant *Klebsiella pneumoniae*. Emerg. Microbes Infect..

[11] Nir-Paz R., Gelman D., Khouri A., Sisson B.M., Fackler J., Alkalay-Oren S., Khalifa L., Rimon A., Yerushalmy O., Bader R. (2019). Successful Treatment of Antibiotic-resistant, Poly microbial Bone Infection With Bacteriophages and Antibiotics Combination. Clin. Infect. Dis..

[12] Kortright K.E., Chan B.K., Koff J.L., Turner P.E. (2019). Phage Therapy: A Renewed Approach to Combat Antibiotic-Resistant Bacteria. Cell Host Microbe.

[13] Chan B.K., Turner P.E., Kim S., Mojibian H.R., Elefteriades J.A., Narayan D. (2018). Phage treatment of an aortic graft infected with *Pseudomonas aeruginosa*. Evol. Med. Public Health.

[14] Dedrick R.M., Guerrero-Bustamante C.A., Garlena R.A., Russell D.A., Ford K., Harris K., Gilmour K.C., Soothill J., Jacobs-Sera D., Schooley R.T. (2019). Engineered bacteriophages for treatment of a patient with a disseminated drug-resistant Mycobacterium abscessus. Nat. Med..

[15] Comeau A.M., Glaziou P., Zumla A., Raviglione M. (2007). Phage-Antibiotic Synergy (PAS): Beta-lactam and quinolone antibiotics stimulate virulent phage growth. PLoS ONE.

[16] Abedon S.T. (2019). Phage-Antibiotic Combination Treatments: Antagonistic Impacts of Antibiotics on the Pharmacodynamics of Phage Therapy?. Antibiotics.

[17] Harding E. (2020). WHO global progress report on tuberculosis elimination. Lancet Respir. Med..

[18] Floyd K., Glaziou P., Zumla A., Raviglione M. (2018). The global tuberculosis epidemic and progress in care, prevention, and research: An overview in year 3 of the End TB era. Lancet Respir. Med..

[19] WHO (2019). Global Tuberculosis Report 2019.

[20] Bardarov S., Bardarov S., Pavelka M.S., Sambandamurthy V., Larsen M., Tufariello J., Chan J., Hatfull G., Jacobs W.R. (2002). Specialized transduction: An efficient method for generating marked and unmarked targeted gene disruptions in Mycobacterium tuberculosis, *M. bovis* BCG and *M. smegmatis*. Microbiology.

[21] Kronheim S., Daniel-Ivad M., Duan Z., Hwang S., Wong A.I., Mantel I., Nodwell J.R., Maxwell K.L. (2018). A chemical defence against phage infection. Nature.

[22] Hampton H.G., Watson B.N.J., Fineran P.C. (2020). The arms race between bacteria and their phage foes. Nature.

