# Supplementary material for: Aminoglycoside Antibiotics Inhibit Mycobacteriophage Infection
Source: Antibiotics (Basel). 2020 Oct 19;9(10):714. doi: 10.3390/antibiotics9100714 (PMC7603143; doi:10.3390/antibiotics9100714)
Supplement: Supplementary file 1 [file antibiotics-09-00714-s001.pdf]

antibiotics

Supplementary Materials

Aminoglycoside Antibiotics Inhibit Mycobacteriophage Infection

Zheng Jiang †, Junwei Wei †, Yunxiang Liang, Nan Peng * and Yingjun Li *

State Key Laboratory of Agricultural Microbiology, College of Life Science and Technology, Huazhong Agricultural University, Wuhan 430070, China; 1021644470@webmail.hzau.edu.cn (Z.J.); weijun.wei@foxmail.com (J.W.); fa-lyx@163.com (Y.L.)

† These authors contributed equally to this work.

* Correspondence: nanp@mail.hzau.edu.cn (N.P.); yingjun@mail.hzau.edu.cn (Y.L.)

Materials and Methods

Transduction of mycobacteriophage plasmid

M. smegmatis mc²155::pMV261 was grown in 7H9 to an OD_{600} of 1.0(~6×10^{8} c.f.u. mL^{-1}). Hundred milliliters of the culture were centrifuged and washed 3 times by 10% glycerol and resuspended in 5 ml 10 % glycerol. The phAE159 plasmid were electransformed into *M. smegmatis* mc²155. Cells were mixed with 3 mL of 7H9 top agar (containing 0.75% agar) and with or without 50 μg/mL kanamycin, then plated on 7H10 agar plates with or without 50 μg/mL kanamycin, and then incubated at 30℃ for 3 days. The number of visible plaques were counted. Both 7H9 agar and 7H10 agar were added when the temperature of the medium was below 55℃. Strains and phages used in this work are shown in Table S1, plasmids used in this work are shown in Table S2, and Table S3 showed the oligonucleotides used in this work.

Phage propagation assay

The phage D29 and phAE159 were collected and serial dilutions by MP buffer, then they were spotted onto the lawns of *Mycobcaterium*. Plates were incubated at 37 °C (30 °C for phAE159) overnight and phages were enumerated by counting plaques. These assays were repeated at least three times with similar results and a representative experiment is shown.

Construction of pSTR1

The *aadA* gene was amplified from pCDFDuet-1 plasmid using the primers STR-F and STR-R in Table S3 and cloned into the pMV261 vector. The plasmid was transformed into *M. smegmatis* mc²155 by standard procedures (1). The recombinant strains were selected on 7H10 agar plates complemented with 50 μg/mL streptomycin and 50 μg/mL kanamycin. The positive clone was named mc²155::pSTR1. Then the mc²155::pSTR1 was incubated in 7H10 agar plates complemented with 50 μg/mL streptomycin or 100 μg/mL spectinomycin.

Growth curves analysis of E. coli being infected by phage T7

E. coli strain DH5α was grown overnight with shaking in lysogeny broth (LB) medium at 37 °C. A 2% subculture was prepared in LB medium supplemented with kanamycin (50 μg/mL) and phage T7 was added at a multiplicity of infection of approximately 0.1. Subsequently, the number of DH5α present in cultures was determined by optical density at 600 nm every 2 h for an additional 15 h. Three biological replicates were tested for each group of kanamycin as well as the control, which contained no kanamycin or phage T7.

Pre-incubation of phage phAE159 with kanamycin

Aliquots of phage phAE159 were incubated at 37 °C for 2 h with or without 50 μg/mL kanamycin. The phages were diluted with MP buffer and added into *M. smegmatis* mc^{2}155::pMV261 cells, then the mixture was suspended in molten 3 mL of 7H9 top agar (with/without 50 μg/mL kanamycin) and overlaid onto 7H10 plates pre-added with kanamycin or not. After incubating the plates at 30 °Cfor 2 days, observe the growth of *M. smegmatis* mc^{2}155.

Transmission electron micrograph analysis

M. smegmatis mc^{2}155 was grown in 7H9 medium at 37 °C with shaking, and the cells were harvested until the OD_{600} (optical density at 600 nM) = 0.8. High titer phage D29 were then added into *M. smegmatis* mc^{2}155 at a multiplicity of infection (MOI) of 100. The culture was incubated for 15 minutes with or without 50 μg/mL streptomycin before microscopic observation. Transmission electron microscopy (TEM) grids (Electron Microscopy Sciences CF400-CU) were prepared by drop-coating grids with each group, washing with water and staining with 2% uranyl acetate. Phages were imaged using the HITACHI H-7650 transmission electron microscope in the Microscopy Imaging Laboratory at the Huazhong Agricultural University.

Quantitative PCRs analysis

M. smegmatis mc^{2}155::pMV261 was grown in 7H9 with 10 folds phage D29 and 50 μg/mL streptomycin. Sampling culture fluid and centrifuged supernatant every 1 hour. Quantitative PCR (qPCR) was performed as standard procedures to quantify D29-DNA (2). Each reaction mixture (20 μL/well) contained 10 μL of Hieff qPCR SYBR Green Master Mix No Rox, 1 μL of 10 μM each of the gp69-qpcr-F and gp69-qpcr-R primer pair in Table S3, 7 μL of DNase- and RNase-free sterile water, and 1 μL of the sample or template DNA. Quantitative PCR and monitoring were performed in an ABI Quant Studio 5 System. PCR amplification was performed with an initial pre-incubation at 95 °C for 5 min, followed by 40 cycles of amplification at 95 °C for 10 s, 60 °C for 20 s and 72 °C for 20 s. A 810 bp PCR product was generated using the standard PCR primers gp69-F/R in Table S3 and D29 DNA to construct the standard curve. After purification and determination of the DNA concentration, the linear double-stranded DNA standard was 10-fold serially diluted to obtain a standard series from 1×10^7 to 1×10^1 copies/μL. The copy numbers of the samples were determined by reading off the standard series with the Ct values of the samples.

Supplementary tables

Table S1. Strains and phages used in this work.

Strains and Phages	Reference
M. tuberculosis H37Ra	Gift from Dr Min Yang
M. bovis BCG	Gift from Dr Min Yang
M. smegmatis mc^2155	Gift from Dr Min Yang
E. coli strain DH5α	Laboratory stock
Mycobacteriophage D29	Gift from Prof Jinsong Li
E. coli phage T7	Gift from Yibao Chen
E. coli phage λ	Gift from Prof Hui Jin

Table S2. Plasmids used in this work.

Plasmids	Genotype and features	Reference
pMV261	A *Mycobacterium-E. coli* shuttle vector carrying kanamycin resistance gene	Gift from Dr Hua Zhang
pRH2521	A *Mycobacterium-E. coli* shuttle vector carrying hygromycin resistance gene	Gift from Dr Min Yang
pSTR1	Derived from pMV261， carrying aminoglycoside adenylyltransferase, confers resistance to streptomycin and spectinomycin	This work
pET28a	A *E. coli* expression vector carrying kanamycin resistance gene	Laboratory stock
phAE159	A mycobacteriophage vector derived from TM4	(3), Gift from Prof Chen Tan

Table S3. Oligonucleotides used in this work.

Oligonucleotide	Sequence (5′-3′)
STR-F	GCAATGGCCAAGACAATTGCGGATATGAGGGAAGCGGTGATCG
STR-R	GCCTGCTGATGATGTCTTAATTAAGGATCTTATTTGCCGACTACCTTGGTGA
gp69-qpcr-F	AGACCGGCGACTACTTCATGG
gp69-qpcr-R	GCAACGGGTCGAACATCGAG
gp69--F	GTGACGCAGATCAAGCTTCC
gp69--R	TCACTTAAAAACGGGGCAACTG

Supplementary figures

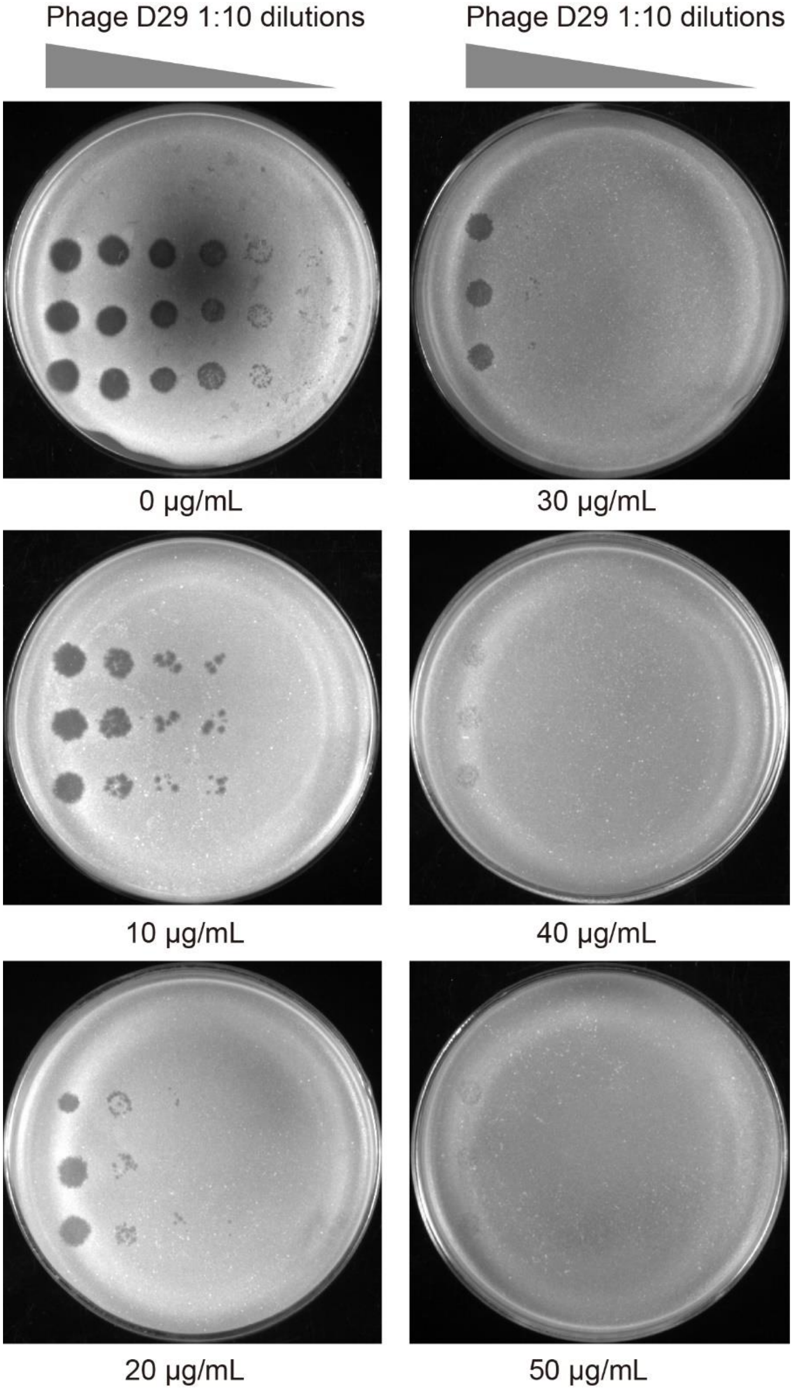

Figure S1. Effects of different concentrations of kanamycin on the infection of *M. smegmatis* mc^{2}155 by phage D29.

Phages were gradually 10-fold diluted from left to right, three replicates on each plate and three biological replicates were performed.

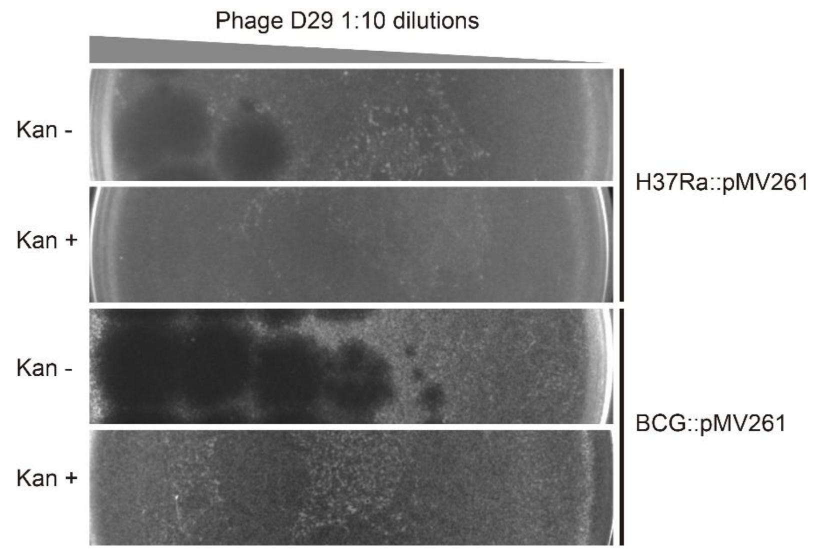

Figure S2. Kanamycin inhibit the infection of *M. tuberculosis* H37Ra and *M. bovis* BCG by phage D29.

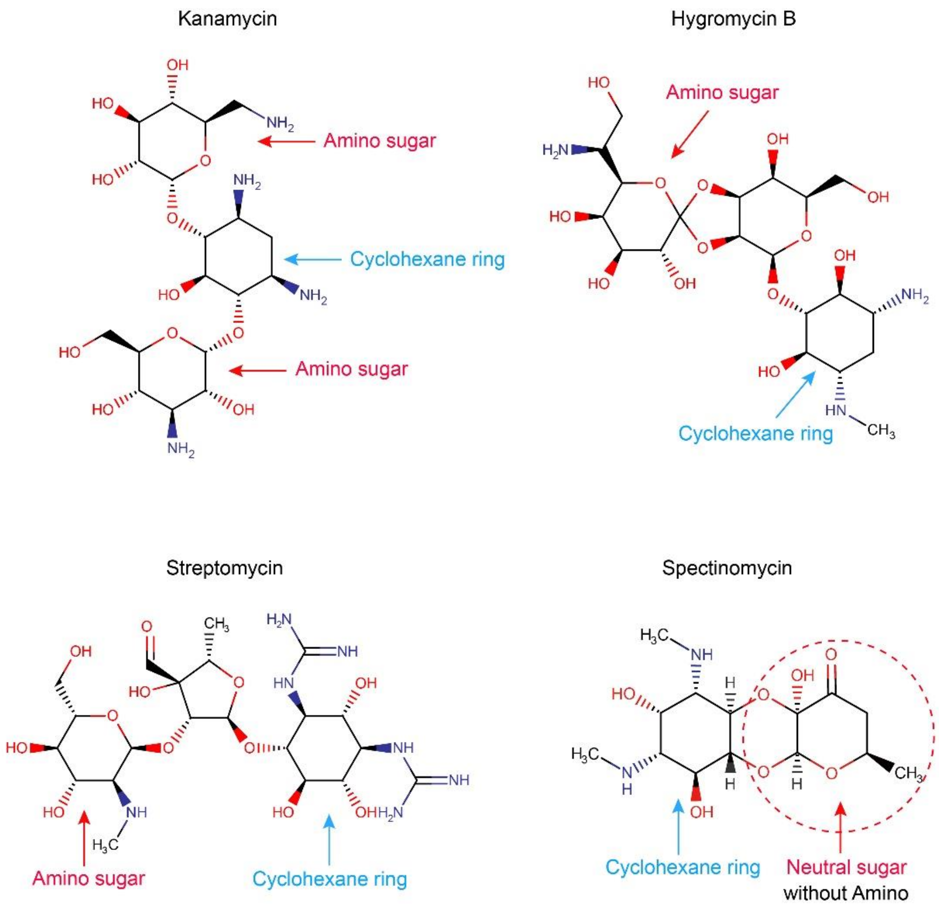

Figure S3. The structures of the four aminoglycoside antibiotics used in this study.

Amino units are in Blue, and Cyclohexane ring and amino sugar are marked with cyan-blue and red arrows, respectively. The neutral sugar without amino in spectinomycin is marked with red dotted circle.

References

1. Li, J.M., Li, N., Zhu, D.Y., Wan, L.G., He, Y.L. and Yang, C. (2008) Isocitrate lyase from Mycobacterium tuberculosis promotes survival of Mycobacterium smegmatis within macrophage by suppressing cell apoptosis. *Chin Med J (Engl)*, **121**, 1114-1119.
2. Miyajima, Y., Satoh, K., Uchida, T., Yamada, T., Abe, M., Watanabe, S., Makimura, M. and Makimura, K. (2013) Rapid real-time diagnostic PCR for Trichophyton rubrum and Trichophyton mentagrophytes in patients with tinea unguium and tinea pedis using specific fluorescent probes. *J Dermatol Sci*, **69**, 229-235.
3. Bardarov, S., Bardarov, S., Jr., Pavelka, M.S., Jr., Sambandamurthy, V., Larsen, M., Tufariello, J., Chan, J., Hatfull, G. and Jacobs, W.R., Jr. (2002) Specialized transduction: an efficient method for generating marked and unmarked targeted gene disruptions in Mycobacterium tuberculosis, M. bovis BCG and M. smegmatis. *Microbiology*, **148**, 3007-3017.
